# ColXV Aggravates Adipocyte Apoptosis by Facilitating Abnormal Extracellular Matrix Remodeling in Mice

**DOI:** 10.3390/ijms21030959

**Published:** 2020-01-31

**Authors:** Tianyu Xia, Zhentong Shen, Jiarui Cai, Miao Pan, Chao Sun

**Affiliations:** College of Animal Science and Technology, Northwest A&F University, Yangling 712100, China

**Keywords:** Collagen XV, adipocytes, apoptosis, extracellular matrix remodeling, fibroblast growth factor 2

## Abstract

The extracellular matrix (ECM) is a highly dynamic structural network and plays an essential role in cell behavior and regulation during metabolic homeostasis and obesity progression. Abnormal ECM remodeling impairs adipocyte plasticity required for diverse cellular functions. Collagen XV (ColXV) is a proteoglycan localized to the outermost layer of basement membranes (BMs) and forms a bridge between the BMs and the fibrillar collagen matrix. Nevertheless, how ColXV affects ECM composition and the reason for subsequent adipocyte apoptosis is still unclear. This report found, through RNA-seq data, that ColXV is linked to cell growth and ECM remodeling. Findings show that, in response to excessive expression of extracellular ColXV, the AMPK/mTORC1 pathway is strongly activated and triggers a cascade of mitochondrial apoptosis. This is the first study to make use of ECM three-dimensional reconstruction, based on decellularization in the adipose tissues and the study reveals that ColXV is an activation factor that alters ECM remodeling in adipose tissues. It was also demonstrated that the fibroblast growth factor 2 (FGF2)/fibroblast growth factor receptor 1 (FGFR1) axis involved in ECM remodeling is suppressed by ColXV due to reduction of FGF2 translocation to FGFR1. Furthermore, ColXV induced remodeling of ECM preceding apoptosis and continued to induce apoptosis in adipocytes. Collectively, our findings establish ColXV as a basement membrane collagen with homology to ColXVIII, indicating that it is one of the positive regulators for inducing ECM remodeling and further promoting adipocyte apoptosis.

## 1. Introduction

The extracellular matrix (ECM) is a highly dynamic three-dimensional structure, continuously undergoing remodeling processes wherein ECM components are degraded, deposited, or otherwise modified. The ECM undergoes remodeling by cells in response to a variety of external and internal stimuli in adipocytes. It especially undergoes a strong structural remodeling when fat mass increases, as seen in obesity [1]. In previous studies, it has been reported that over the course of obesity development, increased interstitial and peripheral ECM deposition in adipose tissues decreases matrix flexibility and reduces tissue plasticity, ultimately leading to dysfunction in adipocytes. For instance, abnormal collagen deposition is a trait for the development of adipose tissue fibrosis and is strongly concerned with tissue inflammation, characterized by macrophage infiltration [2]. Lack of collagen VI (Col6a1) exhibited reduced cell apoptosis accompanied by enlarged adipocytes in ob/ob mice. Furthermore, reduced adipocyte death in these mice was connected with a significant decrease in the spliced form of Xbp1, an endoplasmic reticulum stress marker that causes apoptosis through activation of CHOP and JNK [3]. The volume and number of adipocytes in adipose tissues, which highly depend on the ECM remodeling and apoptosis processes, determine obesity. Despite this, the intrinsic interaction between ECM remodeling and apoptosis still needs to be further investigated.

ColXV is a component of basement membranes which is characterized by extensive interruptions in its collagenous sequences, prompting the name MULTIPLEXIN [4]. Lack of ColXV resulted in defective microvascular hemodynamics and enhanced permeability, in addition to distinct early onset and age-dependent defects in heart structure and function. The impaired structure increased tissue stiffness and was accompanied by a poorly organized fibrillar collagen matrix, in addition to irregularly organized cardiomyocytes with marked interstitial deposition of nonfibrillar protein aggregates [5,6]. This implies that lack of ColXV induces the remodeling of the ECM of cardiomyocytes and causes damage to heart functions after cardiovascular stress. In addition, ColXV is highly homologous with type XVIII collagen in its primary structure. These two have thrombospondin-1 sequence homology in the N-terminus, several homologous collagenous domains, and highly homologous C-terminal non-collagenous domains [7,8]. Type XVIII collagen is the precursor of endostatin, and has been extensively studied for having a potent antiangiogenic effect. It is able for ColXV to suppress angiogenesis and tumor growth by restraining endothelial cell proliferation and promoting endothelial cell apoptosis [8,9]. In adipose tissues, both ColXV and ColXVIII exhibit the ability to regulate adipocyte differentiation [10,11]. After reviewing the previous literature, we found that the mechanisms by which ColXV, a homologous molecule of ColXVIII, regulates adipocytes apoptosis and ECM remodeling still remain elusive.

Objectives In this study, we intended to investigate the effects of ColXV on apoptosis as well as the ECM remodeling of adipocytes. We tried to seek for the signaling pathway by which ColXV promoted apoptosis-like AMPK/mTORC1 signaling pathway. We wanted to further explore the specific mechanisms how ColXV played a key role in adipocyte ECM remodeling to discuss whether we need to take ColXV as a crucial part of adipocyte apoptosis. What’s more, we hoped to in-depth investigate the relationship between ECM remodeling and adipocyte apoptosis.

Thus, our findings displayed relevant mechanisms of ColXV, apoptosis and ECM remodeling, showing potential for the development of novel therapies for obesity and fibrosis.

## 2. Results

### 2.1. ColXV Involved ECM Remodeling and Triggered Apoptosis in Adipocytes

**Figure 1:**
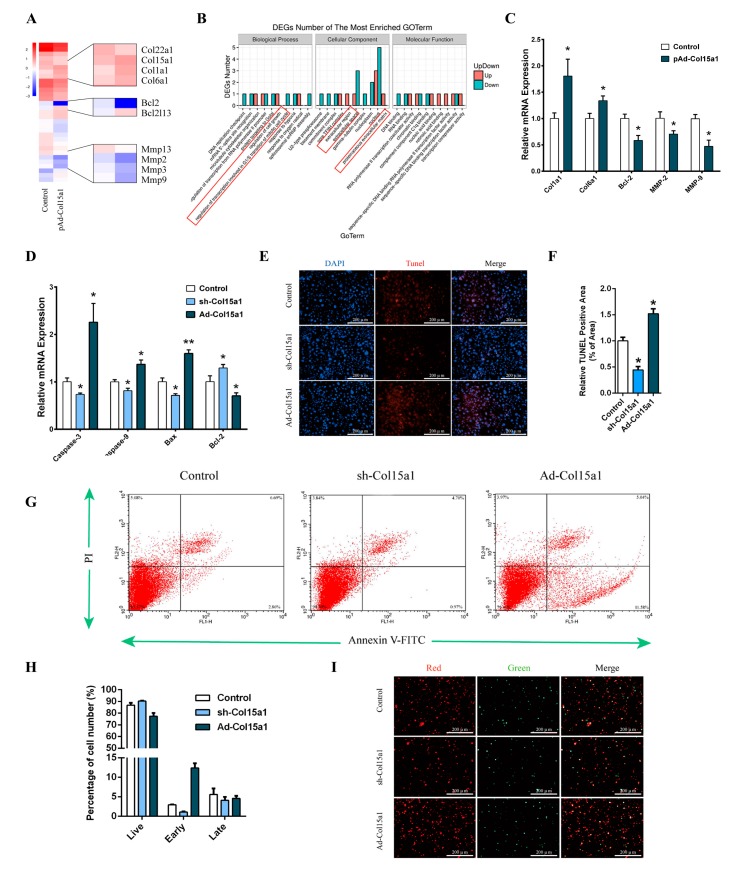
Collagen XV (ColXV) involved extracellular matrix (ECM) remodeling and triggered apoptosis in adipocytes. (**A**) Heatmap of genes set from RNA-seq data with forced expression of ColXV in mice adipocytes, along with the top affected genes (*n* = 4). (**B**) Gene ontology (GO) analysis of the altered genes in (**A**) (*n* = 3). (**C**) Changes in the mRNA level of genes associated with apoptosis and ECM remodeling, which were significantly altered from RNA-seq analysis (*n* = 4). (**D**) Relative mRNA expressions of Caspase-3, Caspase-9, Bax, and Bcl-2 in mice adipocytes in different groups (*n* = 4). (**E**–**F**) Tunnel staining of DNA fragments in adipocytes. Scale bar, 100 μm (*n* = 4) (**G**–**I**) Annexin V-FITC/PI double staining and flow cytometry analysis of adipocytes apoptosis. Scale bar, 100 μm (*n* = 4). Values are presented as mean ± SEM. *p < 0.05, ** *p*< 0.01.

To explore the potential effect of ColXV on adipose tissues, RNA-seq was performed to compare adipocyte transcriptomes treated with Col15a1 overexpression vector or the control. Overexpression of Col15a1 resulted in changes in the expression of signature genes for cell growth and in the composition of the extracellular membrane. Significant differences in gene expression were diagrammatically displayed as a heatmap (Figure 1A). Further analyses revealed that alteration of the expression of signature genes by ColXV was associated with B-cell lymphoma family and ECM remodeling, especially Bcl-2, Col1a1, Col6a1, MMP-2 and MMP-9 (Figure 1A,B). The mRNA expression of the top affected genes in the RNA-seq samples was further examined by quantitative PCR. The results showed that apoptotic factor Bcl-2 was reduced by forced Col15a1. Additionally, the ECM remodeling-related genes Col1a1 and Col6a1 were enhanced by forced Col15a1. Simultaneously, MMP-2 and MMP-9, which are activators of ECM dynamic changes, were also decreased by forced Col15a1 (Figure 1C). These findings showed that ColXV was closely linked to apoptosis and ECM remodeling.

To investigate the role of ColXV in adipocyte growth, we studied the effects of ColXV on different characteristics during the process of adipocyte apoptosis. We transfected adipocytes with adenovirus interference vector of ColXV (sh-Col15a1) or adenovirus overexpression vector of ColXV (Ad-Col15a1). There were no significant differences in the expression of ColXV among different control groups (Figure S1A). Compared with control groups, Ad-Col15a1 treatment showed higher levels of caspase-9, caspase-3 and Bax, and lower levels of Bcl-2 (Figure 1D). TUNEL staining showed increased fragments of genomic DNA in the nucleus after Ad-Col15a1 treatment (Figure 1E,F). To determine whether there was a defect in adipocyte cellular outcomes (apoptosis or survival), Annexin V/PI staining was performed and visualized by flow cytometry. Compared with the control groups, overexpression of Col15a1 induced an increased proportion of apoptotic cells in the early stage, indicating that Ad-Col15a1 is responsible for the change from survival to apoptosis in adipocytes (Figure 1G-I). In summary, our research findings reported that excessive accumulation of ColXV is a driving force to apoptosis in adipocytes.

ColXV exacerbated apoptosis in unfavorable state adipocytes and involved a mitochondrial pathway

**Figure 2:**
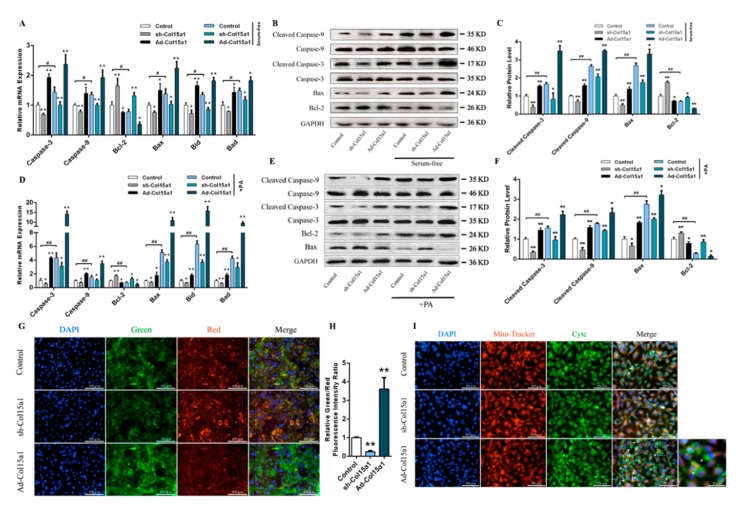
ColXV exacerbated apoptosis in unfavorable state adipocytes and involved a mitochondrial pathway. (**A**–**C**) Relative mRNA and protein expressions of Caspase-3/Cleaved Caspase-3, Caspase-9/Cleaved Caspase-9, Bax, Bcl-2, Bid, and Bad in mice adipocytes in the Control group, sh-Col15a1 group and Ad-Col15a1 group with or without FBS (Fetal Bovine Serum) treatment (*n* = 4). (**D**–**F**) Relative mRNA and protein expressions of Caspase-3/Cleaved Caspase-3, Caspase-9/Cleaved Caspase-9, Bax, Bcl-2, Bid, and Bad in mice adipocytes in the Control group, sh-Col15a1 group and Ad-Col15a1 group with or without PA (palmitate) treatment (*n* = 4). (**G–H**) JC-1 staining of mitochondrial membrane potential in adipocytes. Scale bar, 100 μm (*n* = 4). (**I**) Representative images and enlarged image (bottom right) of Mito-tracker staining (Red) and Cytc (Cytochrome C) immunofluorescent staining (Green) in adipocytes in different groups. Scale bar, 100 μm (*n* = 4). Arrowheads indicate Cytc released from mitochondria to the cytoplasm. Scale bar, 20 μm. Values are presented as mean ± SEM. *p < 0.05, ** *p* < 0.01.

To further determine what features of ColXV are associated with apoptosis, two distinct models of apoptosis were used (treatment with 250 nM palmitate or serum-free medium for 24 h) [12,13,14]. In this study, it was hypothesized that both adverse conditions could effectively induce apoptosis in adipocytes. Overexpression of ColXV caused an increase in Caspase-3, Caspase-9, and the mitochondrial apoptosis markers Bax, Bid and Bad gene expression. However, it caused a decrease in Bcl-2 expression (Figure 2A,D). Western blot analysis confirmed the proapoptotic effect by observing the effects of Ad-Col15al treatment on cleaved caspase-3, cleaved caspase-9, Bax and Bcl-2 expression. The proapoptotic effect with Ad-Col15a1 treatment was also proved by cleaved caspase-3, cleaved caspase-9, Bax and Bcl-2 protein levels (Figure 2B,C,E,F). Since mitochondria were considered the central executioner of apoptosis, we observed the membrane potential of mitochondria and the amount of Cytochrome C (Cytc) released into the cytoplasm by JC-1 staining and immunolocalization respectively [15]. As expected, Ad-Col15a1 led to a significantly higher percentage of green/red fluorescence intensity, indicating that ColXV is capable of inducing a lower mitochondrial membrane potential (Figure 2G,H). Similar to its effect on mitochondrial membrane potential, Ad-Col15a1 treatment also increased the shift from punctate mitochondrial localization to diffuse cytoplasmic Cytc staining (Figure 2I). These observations revealed that after exposure to excessive accumulation of ColXV, adverse state adipocytes display impaired viability and a perturbed mitochondrial membrane structure.

### 2.2. The AMPK/mTORC1 Signaling Pathway was Activated and Acted as a Checkpoint during ColXV Activation of Adipocyte Apoptosis

**Figure 3:**
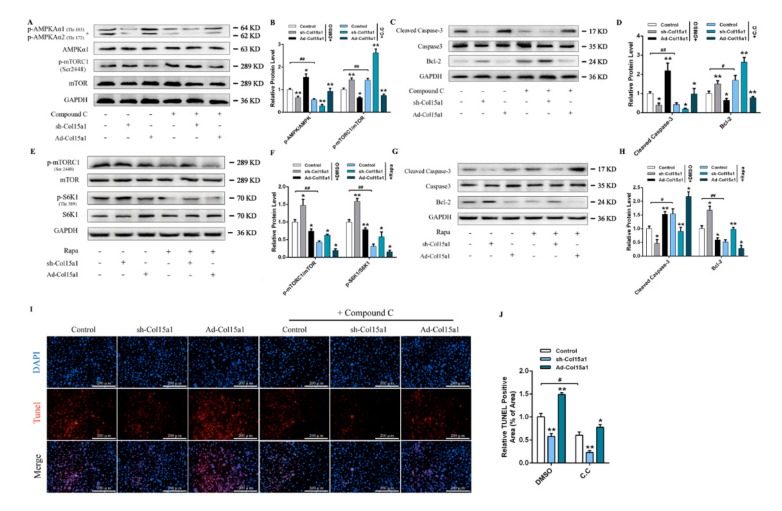
AMPK/mTORC1 signaling pathway was activated and act as a checkpoint during ColXV activating adipocytes apoptosis. (**A**–**B**) Relative protein phosphorylation levels of AMPKAα1^Thr183^, AMPKAα2^Thr172^ and mTORC1^Ser2448^ in different groups with or without Compound C (C.C) treatment (*n* = 3). (**C**–**D**) Relative protein levels of Caspase-3/Cleaved Caspase-3 and Bcl-2 in different groups with or without Compound C (C.C) treatment (*n* = 3). (**E**–**F**) Relative protein phosphorylation levels of mTORC1^Ser2448^ and S6K1^Thr389^ in different groups with or without rapamycin (Rapa) treatment (*n* = 3). (**G**–**H**) Relative protein levels of Caspase-3/Cleaved Caspase-3 and Bcl-2 in different groups with or without rapamycin (Rapa) treatment (*n* = 3). (**I**–**J**) Tunnel staining of DNA fragments in adipocytes with or without Compound C (C.C) treatment. Scale bar, 100 μm (*n* = 4). Values are presented as mean ± SEM. *P < 0.05, ** *P* < 0.01.

It is known that cellular energy sensor AMP-activated protein kinase (AMPK) plays a key role in maintaining adipocyte energy balance and induces energy expenditure in adiposity, with immediate significance for obesity treatment [16,17]. The mammalian target (mTOR) of rapamycin was found to be an important downstream target of AMPK. AMPK/mTOR combine as an energy checkpoint to regulate the synthesis of biomacromolecule compounds and apoptosis via multiple mechanisms, including phosphorylation of S6 kinase [18]. This reminded us to investigate whether ColXV was involved in the regulation of the AMPK/mTOR pathway in adipocytes. We found that ColXV enhanced AMPKAα1^Thr183^ and AMPKAα2^Thr172^ phosphorylation while weakening mTORC1^Ser2448^ and ribosome protein subunit 6 kinase 1 (S6K1^Thr389^) phosphorylation which showed that AMPK/mTORC1 signaling and downstream target S6K1 were disturbed by ColXV (Figure 3A, E). In addition, ColXV promoted the activation of caspase-3 and reduced the protein expression of Bcl-2, accompanied by inactivation of mTORC1 signaling pathway (Figure 3C,G). In this study, the pathway with AMPK inhibitor Compound C (C.C) was further investigated, showing that ColXV significantly reduced AMPK phosphorylation and increased the phosphorylation level of downstream target mTORC1^Ser2448^. It also increased the expression of Bcl-2, in addition to weakening the activity of cleaved caspase-3 (Figure 3A-D). TUNEL staining was also verified by ColXV-induced apoptosis under C.C treatment (Figure 3I,J). Furthermore, adipocytes were treated with rapamycin, a specific phosphorylation inhibitor of mTORC1. We observed lower phosphorylation levels of mTORC1^Ser2448^ and S6K1^Thr389^, accompanied by increased activity of cleaved caspase-3 and reduced protein level of Bcl-2 (Figure 3E–H). Importantly, we found forced ColXV promoted phosphorylation of AMPK and inhibited phosphorylation of mTORC1, while inducing apoptosis of adipocytes, either under C.C treatment or rapamycin treatment. Taken together, these findings provide strong evidence to suggest that ColXV accelerates adipocyte apoptosis via the AMPK/mTORC1/S6K1 signaling pathway.

### 2.3. ColXV Raised Excessive Deposition of ECM Composition in WAT

**Figure 4:**
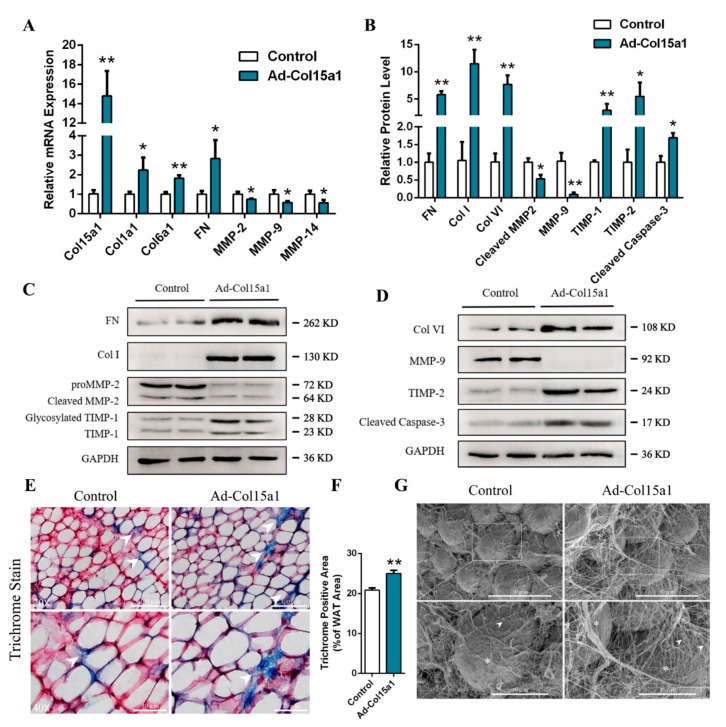
ColXV raised excessive deposition of ECM composition in WAT. All mice were pretreated with either Ad-GFP or Ad-Col15a1. (**A**) Relative mRNA expressions of Col15a1, Col1a1, Col6a1, Fibronectin, MMP-2, MMP-9 and MMP-14 in mice inguinal white adipose tissue (iWAT) (*n* = 6). (**B**–**D**) Relative protein expressions of Fibronectin, Col I, Col VI, MMP-2, MMP-9, TIMP-1, TIMP-2 and Cleaved Caspase-3 in mice iWAT (*n* = 6), each group has two technical repeats. (**E**–**F**) Representative images of Masson’s trichrome staining (collagen is shown as blue, nuclei as black and cytoplasm as red) in frozen-section iWAT samples. Scale bar, 100 μm (top) and 30 μm (bottom) (*n* = 4). Arrowheads indicate thinner collagen fibers (left) and thicker collagen fibers (right). (**G**) Representative images of collagen bundles microstructure on adipocytes surface by scanning electron microscope. Scale bar, 30 μm (top) and 10 μm (bottom) (*n* = 4). Arrowheads indicate thinner collagen bundles (left) and thicker collagen bundles (right). Asterisks indicate adipocytes. Values are presented as mean ± SEM. *p < 0.05, ** *p* < 0.01.

The adipocytes’ niche can be severely perturbed by excessive deposition of adipose tissue, along with aberrant deposition of ECM and altered cell dynamics [19]. To elucidate the effect of ColXV on this niche, we administered Ad-Col15a1 and Ad-GFP to mice white adipose tissue (WAT). We then detected mRNA levels of collagen type I (*Col1a1*), type VI (*Col6a1*), fibronectin (*FN*), *MMP-2*, *MMP-9,* and *MMP-14*, which were shown to be highly expressed in ECM compositions (Col I, Col VI, FN) and have lower expression in matrix metalloproteinases (MMP-2, MMP-9, MMP-14) (Figure 4A). Simultaneously, the protein level of these compositions showed similar changes, accompanying the increased inhibitors of MMPs (TIMP-1, TIMP-2) (Figure 4B–D). Histologically, Masson trichrome staining and scanning with the electron microscope both displayed a significant increase in collagen deposition and distinct distribution patterns of interstitial collagen in mice WAT. In the latter, fibrous regions appeared denser and thicker, and gave rise to larger, bundled collagen fibers (Figure 4E–G).

### 2.4. ColXV Induced ECM Remodeling Dependent on MMPs

**Figure 5:**
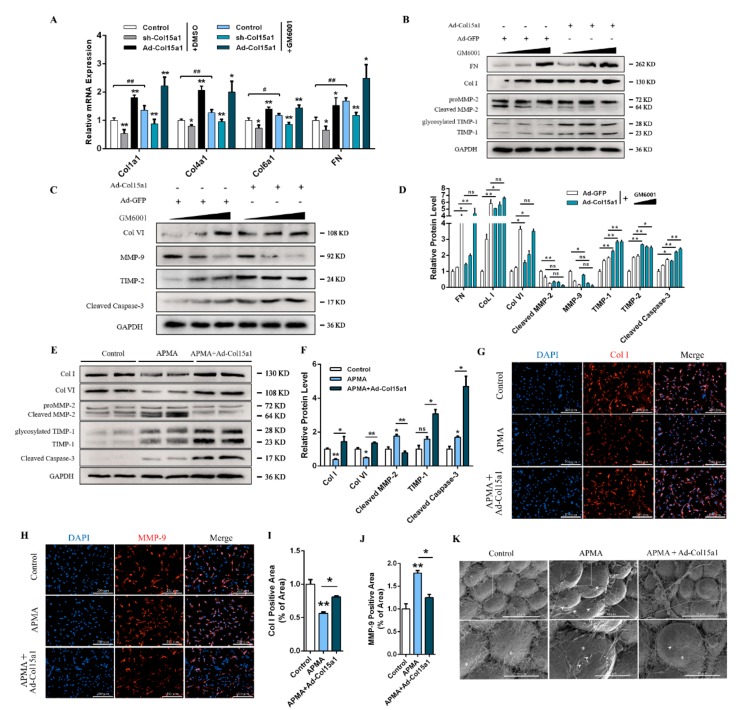
ColXV induced ECM remodeling dependent on MMPs. All mice were pretreated with either Ad-GFP or Ad-Col15a1. (**A**) Relative mRNA expressions of Col1a1, Col4a1, Col6a1 and Fibronectin in mice inguinal white adipose tissue (iWAT) with or without GM6001 (*n* = 6). (**B**–**D**) Relative protein expressions of Fibronectin, Col I, Col VI, MMP-2, MMP-9, TIMP-1, TIMP-2 and Cleaved Caspase-3 in mice iWAT with gradients concentration of GM6001 (*n* = 6). (E-F) Relative protein expressions of Col I, Col VI, MMP-2, TIMP-1 and TIMP-2 in the Control group, APMA group and co-treatment of Ad-Col15a1 and APMA group in mice iWAT (*n* = 6), each group has two technical repeats (**E**). (**G**–**J**) Representative images of Col I and MMP-9 in adipocytes by immunofluorescent staining in different groups (*n* = 4). (**K**) Representative images of collagen bundles microstructure on adipocytes surface by scanning electron microscope. Scale bar, 30 μm (top) and 10 μm (bottom) (*n* = 4). Arrowheads indicate disrupted collagen fibers and exposed adipocytes surface (middle). Asterisks indicate adipocytes. Values are presented as mean ± SEM. *p < 0.05, ** *p* < 0.01, ns, not significant.

To further explore the effects of MMPs on the production and accumulation of ECM components in ColXV-regulated adipocytes, mice were treated with Ilomastat (GM6001), an MMP-specific inhibitor. The mRNA and protein expression levels of ECM-associated components were measured after treatment with Ad-Col15a1. As shown in Figure 5A, the mRNA levels of Col1a1, Col4a1, Col6a1 and FN in adipocytes increased significantly after GM6001 treatment in ColXV overexpressed groups compared with the control. Treating adipocytes with different concentrations of GM6001 (1 nM, 10 nM, and 50 nM) to explore the role of major MMPs in ColXV-induced adipocyte ECM production and accumulation. The results showed a positive correlation between the accumulation of FN, Col I, Col VI, and the increase of GM6001 concentration. The accumulation of MMP-9 and cleaved MMP-2 protein was negatively correlated with the increase in GM6001 concentration. This is consistent with existing findings [20]. More importantly, compared with the control group, forced Col15a1 indistinctively altered the expression of FN, Col I, Col VI, MMP-2 and MMP-9 when the concentration of GM6001 reached 50 nM (Figure 5B–D). It revealed that passivated MMPs impaired the effect of ColXV on the accumulation of ECM components.

Aminophenylmercuric acetate (APMA) is a matrix metalloproteinase activator that was co-treated with Ad-Col15a1 to verify the effects of ColXV on MMP-induced ECM remodeling. As shown in Figure 5E, APMA significantly promoted the activation of MMP-2 and was accompanied by the proteolysis effect of Col I and Col VI. Meanwhile, ColXV reversed APMA-induced activation of MMP-2 and decreased levels of Col I and Col VI. Similar results were obtained by immunofluorescence staining for Col I and MMP-9 (Figure 5G–J). Electron microscopy was used to image the surface fibrous structure of mice adipose tissue. APMA treatment resulted in looser collagen fibers on the surface of adipocytes. The network structure was damaged and exposed to the smooth cell surface, while ColXV treatment relieved reticular fiber surface erosion of adipocytes (Figure 5K). Taken together, ColXV inhibits the activation of major metalloproteinases and promotes the production and accumulation of ECM, with this process depending on MMPs.

### 2.5. Z-stack Analysis and Three-Dimensional Reconstruction of Adipose Tissue ECM under Decellularized Conditions

**Figure 6:**
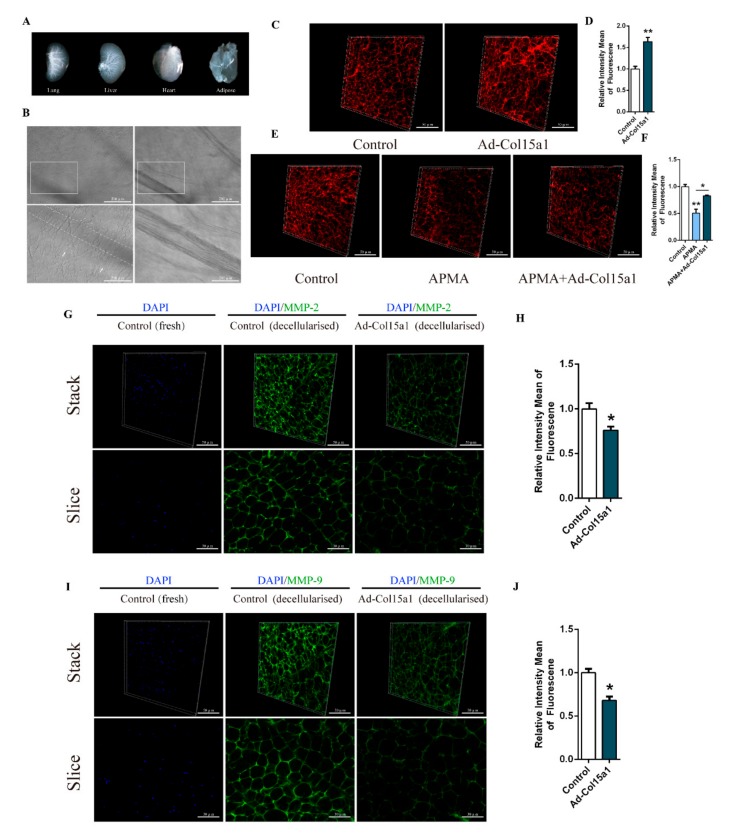
Z-stack analysis and three-dimensional reconstruction of adipose tissue ECM under decellularized conditions. (**A**) Images of decellularized lung, liver, heart and iWAT. (**B**) Representative images of ECM structural integrity and transparency detection in decellularized iWAT. Scale bar, 100 μm (top) and 30 μm (bottom) (*n* = 4). White rectangle represents the images of different focal lengths at the same position (left and right). Arrowheads indicate collagen bundles and right images indicate complete microvascular structure in decellularized iWAT. (**C**–**D**) Representative images of the 3-dimensional structure by Col I immunofluorescent staining of two different groups in decellularized iWAT. Scale bar, 50 μm (*n* = 4). (**E**–**F**) Representative images of the 3-dimensional structure by Col VI immunofluorescent staining of three different groups in decellularized iWAT. Scale bar, 50 μm (*n* = 4). (**G**–**H**) Representative images of the 3-dimensional structure by MMP-2 immunofluorescent staining and DAPI staining of two different groups in decellularized iWAT (two columns on the right). DAPI staining in un-decellularized iWAT(left). Scale bar, 50 μm (*n* = 4). Slice (bottom) represents one of the images composed of the Stack. Scale bar, 30 μm (*n* = 4). (**I**–**J**) Representative images of the 3-dimensional structure by MMP-9 immunofluorescent staining and DAPI staining of two different groups in decellularized iWAT (two columns on the right). DAPI staining in un-decellularized iWAT(left). Scale bar, 50 μm (*n* = 4). Slice (bottom) represents one of the images composed of the Stack. Scale bar, 30 μm (n = 4). Values are presented as mean ± SEM. *p < 0.05, ** *p* < 0.01.

High-resolution imaging, combined with tissue decellularization and tissue transparency, is an efficient visualization method for studying tissue structure and physiological changes in recent years. We decellularized different tissues by infusion of detergents, as described in reference to ISDoT, 3DISCO, BABB, etc. [21,22,23]. The integrity of decellularized tissue and DNA content was measured separately. The results showed that the fiber bundle structure and vascular integrity in the ECM were well preserved, and the DNA in the tissue was almost completely removed (Figure 6A,B, Figure S2A). We performed a Z-stack scan using a high-speed dial laser confocal microscope, followed by three-dimensional reconstruction and ECM fiber diameter analysis. We also performed staining on normal tissue sections for comparison. It was shown that forced ColXV significantly increased Col I abundance and fiber diameter in decellularized ECM (Figure 6C,D, Figure S2B–E and Supplementary Videos S1). Similarly, ColXV relieved APMA-induced degradation of ECM component Col VI under the APMA treatment (Figure 6E, F, Figure S2F and Supplementary Videos S2). These results were consistent with the Col I and Col VI immunofluorescent staining on normal tissue sections (Figure S2B,G). Since MMPs are transported out of the cell membrane and exhibited different binding patterns with extracellular matrix molecules, they are the basis for proteolysis and other biological functions [24,25]. Therefore, we next looked at the changes in the content of MMP-2 and MMP-9 in decellularized adipose tissue, which presented with functional MMP-2 and MMP-9. It was observed that decellularized adipose tissue was removed from the cellular contents, and ColXV had significantly decreased the content of MMP-2 and MMP-9 bound to the ECM (Figure 6G–J and Supplementary Videos S3–S4). Taken together, these results indicate that ColXV reduces MMP-2 and MMP-9 levels associated with ECM and promotes abnormal ECM collagen deposition in adipose tissue.

### 2.6. ColXV Promoted ECM Deposition in Adipose Tissue via the FGF2/FGFR1 Axis

**Figure 7:**
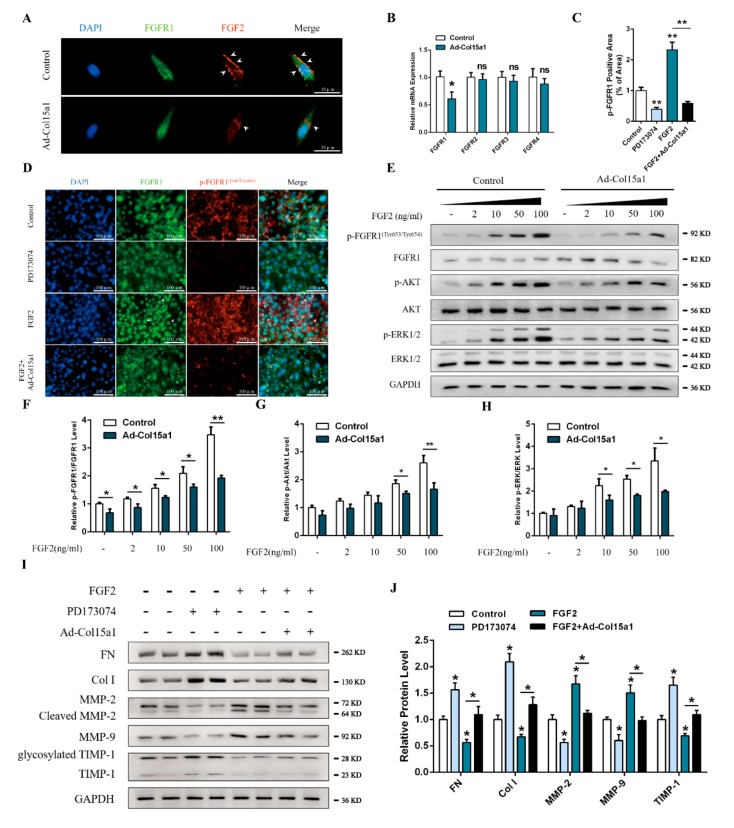
ColXV promoted ECM deposition in adipose tissue via fibroblast growth factor 2 (FGF2)/ fibroblast growth factor receptor 1 (FGFR1) axis. (**A**) Representative images of adipocytes in different groups stained by FGFR1 (green) and FGF2 (red). Arrowheads indicate FGF2 enriched to the cell membrane. Scale bar, 10 µm. (**B**) Relative mRNA levels of FGFR1, FGFR2, FGFR3, and FGFR4 of adipocytes in different groups (*n* = 4). (**C**-**D**) Representative images of adipocytes in different groups were stained by FGFR1 (green) and p-FGFR1^Tyr653/Tyr654^(red). Arrowheads indicate FGFR1 transferred into the nucleus. Scale bar, 100 µm (*n* = 4). (**E**–**H**) Relative protein phosphorylation levels of FGFR1^Tyr653/Tyr654^, AKT, and ERK1/2 of two groups in adipocytes with gradients concentration of FGF2 (*n* = 4). (**I**–**J**) Relative protein levels of FN, Col I, MMP-2, MMP-9, and TIMP-1 of adipocytes in different groups (*n* = 4). Values are presented as mean ± SEM. *p < 0.05, ** *p* < 0.01, ns, not significant.

The collagen scaffold sustains the release of growth factors and plays a major role in maintaining the stability of growth factors [26,27,28]. We hypothesized that ColXV might share similar functions to regulate ECM remodeling in adipocytes. To validate this hypothesis, we determined the effect of ColXV on the distribution of extracellular FGF2. As shown in Figure 7A, ColXV treatment reduced FGF2 enrichment to FGFR1 (fibroblast growth factor receptor 1). Importantly, we observed decreased expression of FGFR1, but not FGFR2, FGFR3, or FGFR4, in response to ColXV treatment (Figure 7B). We then examined the FGF2-activated FGFR1^(Tyr653/Tyr654)^ phosphorylation level with FGFR1-specific inhibitor PD173074 and FGF2 murine recombinant protein. PD173074 significantly decreased the phosphorylation intensity of FGFR1^(Tyr653/Tyr654)^. The recombinant FGF2 had the opposite effect, and promoted the translocation of FGFR1 into the nucleus. Meanwhile, overexpression of ColXV effectively reduced the intensity of fluorescence of phosphorylated FGFR1^(Tyr653/Tyr654)^ and inhibited the translocation of FGFR1 into the nucleus (Figure 7C,D). We further examined the phosphorylation levels of the downstream molecules that were activated by FGF2. As shown in Figure 7E, phosphorylation of FGFR1, ERK1/2 and AKT was strengthened with increasing FGF2 concentration, while forced ColXV significantly inhibited phosphorylation levels of FGFR1^(Tyr653/Tyr654)^, ERK1/2 and AKT (Figure 7E–H). We next assessed the relationship between the FGF2/FGFR1 axis and ECM remodeling in adipocytes. The addition of PD173074 inhibited the expressions of cleaved MMP-2 and MMP-9 and increased the abundance of Col I and FN compared with the control group. In contrast, FGF2 showed the opposite effect. Importantly, ColXV significantly inhibited the increase of MMP-2 activity and decreased FGF-2-induced Col I and FN (Figure 7I–J). Collectively, ColXV restrains the FGF2/FGFR1 axis by reducing FGF2 recruitment to the receptor FGFR1, negatively regulating FGF2-induced ECM remodeling.

### 2.7. Abnormal ECM Remodeling Persistently Stimulated Apoptosis of Adipocytes

**Figure 8:**
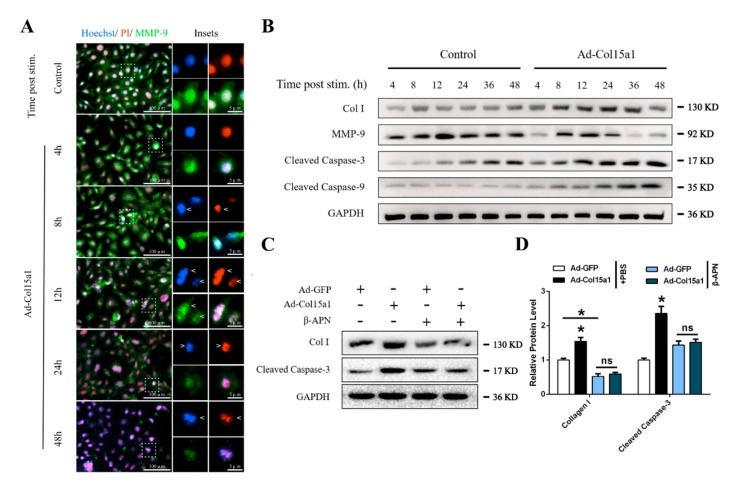
**Abnormal ECM remodeling persistently stimulated apoptosis of adipocytes.** All groups were pretreated with PA to induce adipocytes apoptosis in (**A**) and (**B**). (**A**) Representative images of adipocytes morphological characteristics in different groups with the extension of stimulation time stained by Hoechst 33258 (blue), PI (red), and MMP-9 (green). Arrowheads indicate the morphological characteristics changes of adipocytes nucleus. From top to bottom represent deformed nucleus, marginalized chromatin and disintegrated nucleus, respectively. Scale bar, 100 µm (left) and 5 µm (right). (**B**) Relative protein levels of Col I, MMP-9, Cleaved Caspase-3 and Cleaved Caspase-9 of adipocytes in different groups with the extension of stimulation time (*n* = 4). (**C**–**D**) Relative protein levels of Col I and Cleaved Caspase-3 of adipocytes in different groups (*n* = 4). Values are presented as mean ± SEM. *p < 0.05, ** *p* < 0.01, ns, not significant.

ColXV caused adipocyte ECM remodeling and apoptosis, and therefore a correlation between the two required consideration. Hoechst, PI and MMP-9 immunofluorescence staining were performed to evaluate this, and it was found that ColXV induced apoptosis of adipocytes over time. After 4 h of ColXV treatment, a part of the nuclei became brighter, and at 8 h the cell nuclei began to deform. After 12 h, the chromatin was concentrated and marginalized. At 24 h, the nuclei were further shrunken. The nuclei became smaller and disintegrated to form massive apoptotic bodies at 48 h. At the same time, immunofluorescence staining showed that ColXV-induced apoptosis of adipocytes occurred, and the fluorescence intensity of ECM degradation-related factor MMP-9 in adipocytes gradually decreased (Figure 8A). The protein levels of MMP-9, cleaved caspase-3 and cleaved caspase-9 in adipocytes at different times after transfection with ColXV were also detected. Compared with the control group, the expressions of cleaved caspase-3 and cleaved caspase-9 increased with the extension of treatment time, and MMP-9 showed increase firstly before decrease with the extension of treatment time. The trend followed a sharp decline at 12 h, after treatment with ColXV, while collagen type I showed delayed expression along the timeline (Figure 8B). This suggested that ColXV-induced ECM remodeling precedes apoptosis. Interestingly, we found that ECM remodeling induced by ColXV was independent of transforming growth factor β1 (TGFβ1), which is widely regarded as an important factor causing ECM deposition [29,30] (Figure S1B). Next, the collagen synthesis process was blocked with β-APN, an inhibitor of lysyloxidase. As shown in Figure 8C,D, β-APN treatment reduced collagen type I abundance and relieved ColXV-induced apoptosis in adipocytes. Taken together, we find that abnormal ECM deposition induced by ColXV promotes adipocyte apoptosis.

## 3. Discussion

The dynamic changes of ECM and the regulation of the physiological functions of cells depend on their three-dimensional topological structures. The high-resolution imaging of tissue ECM facilitates a more accurate reflection of changes in the ECM physiological processes. However, the tissue thickness, organelles, and cell structures with different refractive powers cause great scattering and dissipation of light in optical imaging. Therefore, making tissues transparent and getting three-dimensional stereo imaging would help improve our understandings of the molecular composition and spatial distribution of the ECM. In previous studies, there were several methods used to enhance tissue transparency and optical imaging depth, such as ISDoT, PACT, CLARITY, Scale/ScaleA2, SeeDB, PARS, ClearT/ClearT2, 3DISCO and BABB [21,22,23,31,32,33,34,35,36]. This study depends on ISDoT, 3DISCO, ClearT and other methods to decellularize tissues and perform three-dimensional reconstruction of the ECM. We used these methods to further verify that ColXV induced ECM remodeling in adipose tissues and reduced MMP-2 and MMP-9 binding to the ECM skeleton.

**Figure 9:**
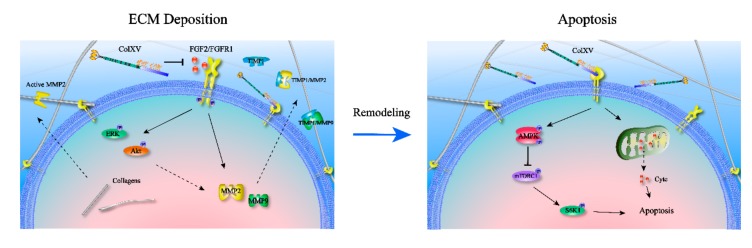
Graphical Abstract: ColXV aggravated apoptosis in mice white adipocytes by facilitating abnormal extracellular matrix remodeling.

Fibroblast growth factors mediate extracellular-to-intracellular signal transduction through a wide range of regulations in various biological processes via paracrine and autocrine signaling. The complex interactions between FGF2 and ECM molecules control the bioavailability, stability, and concentration of FGF2 in the microenvironment [37,38]. FGF2 extensively combines with extracellular heparan sulfate to form a tetrameric complex effectively, which reduces the binding of FGF2 to cellular receptors, while the complex releases FGF2 via protease hydrolysis [39]. It was demonstrated that ColXV reduced FGF2 attachment to the cell surface possibly by inhibiting the protease activity in promoting dissociation of FGF2 from the heparanase complex. FGF2 strongly activates downstream signaling pathways by binding to its receptors, which effectively promote the phosphorylation of AKT, ERK1/2 and other molecules to induce fibroblast proliferation. Studies have shown that FGF2 induces kidney fibrosis [40]. Surprisingly, low molecular weight FGF2 in the liver has been reported to inhibit CCl_4_-induced fibrosis, whereas high molecular weight FGF2 has the opposite effect. The ability of low molecular weight FGF2 to resist excessive fibrosis of cardiomyocytes has also been further validated in the heart [41,42,43]. In addition, FGF2 also showed an inhibitory effect on hypertrophic scar fibrosis, accompanied with upregulation of MMP-1 [44]. Here, we show that ColXV inhibits the translocation of FGF2 with FGFR1 and downstream ERK/Akt signaling to reduce MMP-2 and MMP-9 binding to the ECM skeleton so that impairs FGF2-induced ECM remodeling in adipocytes (Figure 9). Our study demonstrates that ColXV-induced extracellular collagen deposition is associated with the FGF2/FGFR1 axis.

Apoptosis is a biological phenomenon controlled by genes highly conserved in evolution to remove damaged cells. It plays a key role in regulating embryonic development and tissue homeostasis. Cells are stimulated to induce apoptosis by a variety of external and internal factors, such as the absence of growth factors, activation of specific death receptors, radiation, viral infections, and DNA damage. Here, we found that ColXV promoted adipocyte apoptosis via Hoechst/PI staining, Annexin V-FITC/PI staining and TUNEL staining. Importantly, we found that ColXV triggered adipocyte apoptosis through the mitochondrial and AMPK/mTORC1 signaling pathways. AMPK has recently been proposed as a regulator of cell apoptosis or survival under stress conditions while the most well-known functions of AMPK are regulating energy and intracellular homeostasis. AMPK/mTORC1 is the most important metabolic checkpoint for controlling cell death. Based on this process, inhibition of mTORC1 by AMPK activation causes a decrease in the level of intracellular protein translation. A large number of short-lived anti-apoptotic proteins such as MCL1 are reduced, making cells susceptible to mitochondrial apoptosis [45]. In addition, since mTOR inhibits apoptosis through the mediation of p53 and p27, the negative effect of AMPK on mTORC1 also makes cells sensitive to apoptosis [46].

In many studies, apoptosis induced by collagens has mainly focused on the phenomenon of anoikis. The ECM senses changes in mechanical force and sends signals through the integrin receptor into intracellular susceptors. Anoikis is an apoptotic process triggered by abnormal cell adhesion [47]. In addition, the alternative splicing of fragments from collagens is another way to induce apoptosis. Endostatin, an NC1 fragment of ColXVIII, inhibits endothelial cell growth and angiogenesis, and has exhibited the ability to inhibit FGF2-induced bovine capillary endothelial cell proliferation [8,48]. ColXV and ColXVIII are in the same collagen subclass of MULTIPLEXINs and have similar structures. Although studies have reported that ColXV also exhibits the ability to inhibit tumor migration and growth, the NC1 fragment of ColXV has surprisingly not shown similar apoptosis-induced effects [49,50,51]. Together, we demonstrate that ColXV induces apoptosis in adipocytes via the AMPK/mTORC1/S6K1 pathway as well as mitochondrial apoptosis after ECM remodeling (Figure 9).

ECM is a three-dimensional structure produced, assembled and modified by cells, which, in turn, alters cell behavior and maintains the extracellular microenvironment. Through a range of cell-ECM interactions, tissues respond to physiological stresses such as injury, infection, and disease. Aberrant ECM remodeling in which abnormal accumulation or fibrosis of ECM components is the most induces apoptosis or necrosis. In particular, fibrosis of the kidney, liver and heart will seriously affect the normal physiological functions of organs, and is accompanied with the occurrence of apoptosis [52,53]. In adipose tissues, obesity is the main cause of excessive collagen deposition and fibrosis. The new vascular system is not involved in rapid adipose tissue expansion when adipose tissue hypoxia, tissue inflammation, and apoptosis happen [54]. In previously published literature, it has been shown that knocking out collagen type IV in ob/ob mice significantly improves glucose and lipid metabolism, increases insulin sensitivity, and reduces adipose tissue inflammation and cell necrosis. This may be related to lack of collagen V, resulting in enhanced ECM plasticity over the course of obesity development [3]. During obesity-induced fibrosis, a weak TNF-associated inducer of apoptosis (TWEAK) was also found to suppress adipocyte hypertrophy and ECM remodeling, in addition to reducing the occurrence of apoptosis [55]. It suggests that ColXV promotes abnormal accumulation of adipocytes ECM and further induces apoptosis may be related to ColXV altered inherent ECM structure. In summary, we exposed structural element ColXV in adipocytes basement membrane is critical for the maintenance microenvironment homeostasis of adipocytes. Meanwhile, excessive ColXV activates the FGF2/FGFR1 axis and induced abnormal ECM accumulation in adipocytes, ultimately leading to sustained apoptosis (Figure 9). This study may contribute to further understanding of the regulatory mechanisms of adipocyte expansion and apoptosis. As the study proves that adipocyte apoptosis is a process after ECM remodeling, it provides another strong evidence for ECM remodeling-induced apoptosis. Since the fibrosis caused by ColXV-induced ECM remodeling in adipose tissues is highly involved with obesity, this study will facilitate the search for novel approaches for obesity-related therapies. What’s more, in our research, we find that ColXV transmembrane signal transmission and the functions of NC1 hydrolysate are not clear enough, the specific molecular mechanism about how ColXV results in adipocyte apoptosis via ECM remodeling still need to be explored. We also wanted to connect ColXV-ECM remodeling with more obesity phenomenon such as white adipose tissue browning.

## 4. Materials and Methods

### 4.1. Animals

Six-week-old C57BL/6J male mice were purchased from the Laboratory Animal Center of the Fourth Military Medical University (Xi’an, China). Animals were raised and sampled in accordance with the guidelines and regulations (T/NWSUAF, 235-2014, 9 May 2014) approved by the Animal Ethics Committee of the Northwest A&F University (Yangling, China). Recombinant ColXV (Ad-Col15a1) adenovirus overexpression vector and interference vector (sh-Col15a1) were intraperitoneally injected into mice for 2 weeks to study the physiological changes of tissues. For the study of MMP in vivo, iWAT was administered to GM6001 (Ilomastat, Selleck) or APMA (4-Aminophenylmercuric acetate, Sigma–Aldrich) in DMSO for 7 days. iWAT was then sampled for a future study.

### 4.2. Isolation of Adipocytes

Primary adipocytes were isolated and cultured as per a previously described method [56]. In experiments involving the administration of Ad-Col15a1 or sh-Col15a1 (24 h or 48 h at the titer of 1 × 10^9^ IFU/mL), AMPK inhibitor (Compound C, Selleck, Houston, Texas, USA), mTORC1 inhibitor (rapamycin, Selleck), MMP agonist (4-Aminophenylmercuric acetate, Sigma–Aldrich, St. Louis, Missouri, USA), FGFR1 inhibitor (PD173074, Selleck), LOX inhibitor (β-APN, Sigma–Aldrich), or FGF2 recombinant protein (50037-M07E, Sino Biological, Beijing, China), cells were allowed to adhere for 48 h before treatment. The medium was subsequently replaced, and adipocyte petri dishes were treated with the indicated reagents. Cells were assessed by RT-PCR and western blot or fixed with 4% paraformaldehyde and stained for markers of interest.

### 4.3. RNA-Seq Analysis

Inguinal white adipose tissue (iWAT) of 4-week old mice was harvested for use as a source of cells for experimentation. The adipocytes were infected with purified overexpressing Col15a1 adenoviral vector (pAd-Col15a1) or a blank adenoviral vector (control). The total RNA from the adipocytes was infected with different adenovirus vectors prepared with RNAiso reagent (Takara, China, D312), and the RNA-seq analysis was performed as previously described [57].

### 4.4. Scanning Electron Microscopy

The fresh iWAT was cut into small pieces and quickly placed into a pre-cooled 2.5% glutaraldehyde fixative overnight at 4 °C. The samples were then washed three times with PBS (pH 7.4). The cells were post-fixed in 1% osmium tetroxide in the cacodylate buffer for 30 h and washed with buffer three times. Following this, the samples were dehydrated once in each concentration of ethanol for 15 min. Further dehydration of the samples was done with the EM CPD300 automated critical point dryer (Leica, Weztlar, Germany), and samples were sprayed using a Quorum Q150R vacuum magnetron ion sputter coater (Quorum, Nottingham, UK). The samples were imaged with a Nova Nano SEM-450, operating at 5 kV (Thermo Fisher Scientific, Waltham, Massachusetts, USA).

### 4.5. Apoptosis Assessment

Adipocyte apoptosis was measured by FITC-labeled annexin V/PI staining and TUNEL staining. An Annexin V-FITC and TUNEL apoptosis assay kit (Vazyme, Nanjing, China) was used according to the manufacturer’s protocol. The cells were then observed using a Nikon TE2000-U microscope, and the data were analyzed by using Image J software.

### 4.6. Immunofluorescence Analysis

Adipose slices were subjected to microwave antigen retrieval in EDTA or citrate buffer and then gradually cooled. Samples were permeabilized with 0.5% Triton X-100 in PBS for 10 min and blocked with goat serum for 1 h at room temperature. The samples were incubated overnight at 4 °C with the antibody diluted in goat serum blocking solution. Anti-Collagen I (ab138492, Abcam, Cambridge, UK) and anti-Collagen VI (ab6588, Abcam) antibodies were used to identify the changes in ECM composition under normal conditions. Ninety-nine micrometer thick frozen sections of decellularized adipose tissue were blocked with goat serum in 0.1% Triton for 1 h at 37 °C. Then, samples were incubated overnight at 4 °C with Anti-Collagen I (ab138492, Abcam), Anti-Collagen VI (ab6588, Abcam), Anti-MMP9 (ab38898, Abcam) and Anti-MMP2 (ab92536, Abcam) antibodies diluted in goat serum. After three 30 min washes with PBS, samples were stained for 4 h at room temperature with fluorescent secondary antibodies (Abcam), followed by 10 min of DAPI staining for nucleus visualization. Slides were then viewed under a rotary laser confocal microscope (Revolution WD high-speed rotary laser confocal microscopy, Andor). Z-stack analysis was used to continuously image the decellularized ECM structure. Three-dimensional reconstruction was performed with the Imaris 9.0 software.

### 4.7. Cell Culture Immunostaining

Adherent cells were grown on a 96-well plate. The cells were fixed with 4% PFA in PBS for 10 min and permeabilized with 0.2% Triton X-100 in PBS for 5 min. They were blocked with PBS containing 0.1% Triton and then goat serum for 45 min at room temperature. For immunostaining, the cells were incubated for 2 h with the following antibodies diluted in the blocking solution: Anti-Cytochrome C (ab33589, Abcam), Anti-Collagen I (ab138492, Abcam), Anti-MMP9 (ab38898, Abcam), Anti-MMP2 (ab92536, Abcam) antibodies, Anti-FGF2 (ab8880, Abcam), Anti-FGFR1 (ab31324, Abcam) and Anti-pFGFR1 (GTX133526, GeneTex, Irvine, California, USA).

### 4.8. Quantitative Real-Time PCR (qRT–PCR)

Total RNA was isolated using TRIpure reagent kit (Takara, Dalian, China) according to the manufacturer’s protocol. The cDNA used in the experiments was synthesized with the High Capacity cDNA Reverse Transcription Kit (Takara, China) according to the manufacturer’s protocol. qRT-PCR was performed using SYBRGreen PCR Master Mix (Vazyme, Nanjing, China). PCR primers were as follows: Col15a1 Forward: 5′-TCC GAG ATG GTT GGA AAA AG-3′, reverse: 5′-AAA TGG GGT TCA GTG GAG GT-3′; Col1a1 Forward: 5′-GCT CCT CTT AGG GGC CAC T-3′, reverse: 5′-ATT GGG GAC CCT TAG GCC AT-3′; Col6a1 Forward: 5′-CTG CTG CTA CAA GCC TGC T-3′, reverse: 5′-GCA CGA AGA ATA GAT CCA CAG GG-3′; Fibronectin Forward: 5′-ATG TGG ACC CCT CCT GAT AGT-3′, reverse: 5′-ATG TGG ACC CCT CCT GAT AGT-3′; MMP-2 Forward: 5′-CAA GTT CCC CGG CGA TGT C-3′, reverse: 5′-TTC TGG TCA AGG TCA CCT GTC-3′; MMP-9 Forward: 5′-CTG GAC AGC CAG ACA CTA AAG-3′, reverse: 5′-CTC GCG GCA AGT CTT CAG AG-3′; MMP-14 Forward: 5′-CAG TAT GGC TAC CTA CCT CCA G-3′, reverse: 5′-GCC TTG CCT GTC ACT TGT AAA-3′; Caspase-3 Forward: 5′-ATG GAG AAC AAC AAA ACC TCA GT-3′, reverse: 5′-TTG CTC CCA TGT ATG GTC TTT AC-3′; Caspase-9 Forward: 5′-TCC TGG TAC ATC GAG ACC TTG-3′, reverse: 5′-AAG TCC CTT TCG CAG AAA CAG-3′; Bcl-2 Forward: 5′-GTC GCT ACC GTC GTG ACT TC-3′, reverse: 5′-CAG ACA TGC ACC TAC CCA GC-3′; Bax Forward: 5′-TGA AGA CAG GGG CCT TTT TG-3′, reverse: 5′-AAT TCG CCG GAG ACA CTC G-3′; Bid Forward: 5′-GCC GAG CAC ATC ACA GAC C-3′, reverse: 5′-TGG CAA TGT TGT GGA TGA TTT CT-3′; Bad Forward: 5′-AAG TCC GAT CCC GGA ATC C-3′, reverse: 5′-GCT CAC TCG GCT CAA ACT CT-3′; FGFR1 Forward: 5′-TAA TAC CAC CGA CAA GGA AAT GG-3′, reverse: 5′-TGA TGG GAG AGT CCG ATA GAG T-3′; FGFR2 Forward: 5′-CCT CGA TGT CGT TGA ACG GTC-3′, reverse: 5′- CAG CAT CCA TCT CCG TCA CA-3′; FGFR3 Forward: 5′-GCC TGC GTG CTA GTG TTC T-3′, reverse: 5′-TAC CAT CCT TAG CCC AGA CCG-3′; FGFR4 Forward: 5′-GCT CGG AGG TAG AGG TCT TGT-3′, reverse: 5′-CCA CGC TGA CTG GTA GGA A-3′; GAPDH Forward: 5′- AGG TCG GTG TGA ACG GAT TTG-3′, reverse: 5′- TGT AGA CCA TGT AGT TGA GGT CA-3′. The levels of mRNA were normalized using GAPDH. The expressions of genes were analyzed by the method of 2^−ΔΔCt^.

### 4.9. Western Blotting Analysis

Western blotting was performed with the SDS-PAGE electrophoresis system. Adherent cells or adipose tissue extracts were prepared and transferred to PVDF membranes. The following primary antibodies were used: anti-GAPDH (ap0063, Bioworld), anti-Col15α1 (ab150463, Abcam), anti-Caspase-9 (ab32539, Abcam), anti-Cleaved Caspase-9 (bs7070, Bioworld), anti-Caspase-3 (Bs6428, Bioworld), anti-Cleaved Caspase-3 (bs7004, Bioworld), anti-Bcl2 (bs1511, Bioworld), anti-Bax (ab32503, Abcam), anti-AMPK (ab32047, Abcam), anti-pAMPK (ab133448, Abcam), anti-mTOR (ab87540, Abcam), anti-pmTORC1^Ser2448^ (ab109268, Abcam), anti-Akt (ab8805, Abcam), anti-pAkt^Ser473^ (ab18206, Abcam), anti-S6K1 (ab32529, Abcam), anti-pERK1/2 (ab201015, Abcam), anti-ERK1/2 (ab17942, Abcam), anti-pS6K1^Thr389^ (ab2571, Abcam), anti-Collagen I (ab34710, Abcam), anti-Collagen VI (ab6588, Abcam), anti-Fibronectin (ab2413, Abcam), anti-MMP2 (ab92536, Abcam), anti-MMP9 (ab38898, Abcam), anti-TIMP1 (WL02342, Wanleibio), anti-TIMP2 (ab180630, Abcam), anti-FGFR1 (ab31324, Abcam), anti-pFGFR1^Tyr653/Tyr654^ (GTX133526, GeneTex), anti-TGFβ1 (WL03092, Wanleibio). Horseradish peroxidase anti-rabbit or anti-goat (Sigma–Aldrich) were used as secondary antibodies.

### 4.10. Statistical Analysis

Data were analyzed by using one-way ANOVA in SAS v8.0 (SAS Institute, Cary, NC) software. Comparisons between the treatment groups and control were performed by Bonferroni’s multiple comparison tests. Data are presented as mean ± SD, and *p*< 0.05 is considered to be significant.

## Supplementary Materials

The following are available online at https://www.mdpi.com/1422-0067/21/3/959/s1, Figure S1: Carrier expression efficiency and influence analyses. Figure S2: Z-stack analysis and three-dimensional reconstruction. Video S1: Z-stack and three-dimensional reconstruction of Collagen type I in Ad-ColXV group and Control group, Video S2: Z-stack and three-dimensional reconstruction of Collagen type VI in different groups (Control group, APMA group and co-treatment with APMA and Ad-ColXV group), Video S3: Z-stack and three-dimensional reconstruction of MMP-2 in Ad-ColXV group and Control group, Video S4: Z-stack and three-dimensional reconstruction of MMP-9 in Ad-ColXV group and Control group.
