# Supplementary material for: ColXV Aggravates Adipocyte Apoptosis by Facilitating Abnormal Extracellular Matrix Remodeling in Mice
Source: Int J Mol Sci. 2020 Jan 31;21(3):959. doi: 10.3390/ijms21030959 (PMC7037489; doi:10.3390/ijms21030959)
Supplement: Supplementary file 1 [file ijms-21-00959-s001.zip › Supplementary videos/Contents.pdf]

Supplementary Videos

Video 1. Z-stack and and three-dimensional reconstruction of Collagen type I in Ad-ColXV group and Control group.

Video 2. Z-stack and and three-dimensional reconstruction of Collagen type VI in different groups (Control group, APMA group and co-treatment with APMA and Ad-ColXV group).

Video 3. Z-stack and and three-dimensional reconstruction of MMP-2 in Ad-ColXV group and Control group.

Video 4. Z-stack and and three-dimensional reconstruction of MMP-9 in Ad-ColXV group and Control group.
