# Supplementary material for: ColXV Aggravates Adipocyte Apoptosis by Facilitating Abnormal Extracellular Matrix Remodeling in Mice
Source: Int J Mol Sci. 2020 Jan 31;21(3):959. doi: 10.3390/ijms21030959 (PMC7037489; doi:10.3390/ijms21030959)
Supplement: Supplementary file 1 [file ijms-21-00959-s001.zip › Supplementary videos/Supplementary figures.docx]

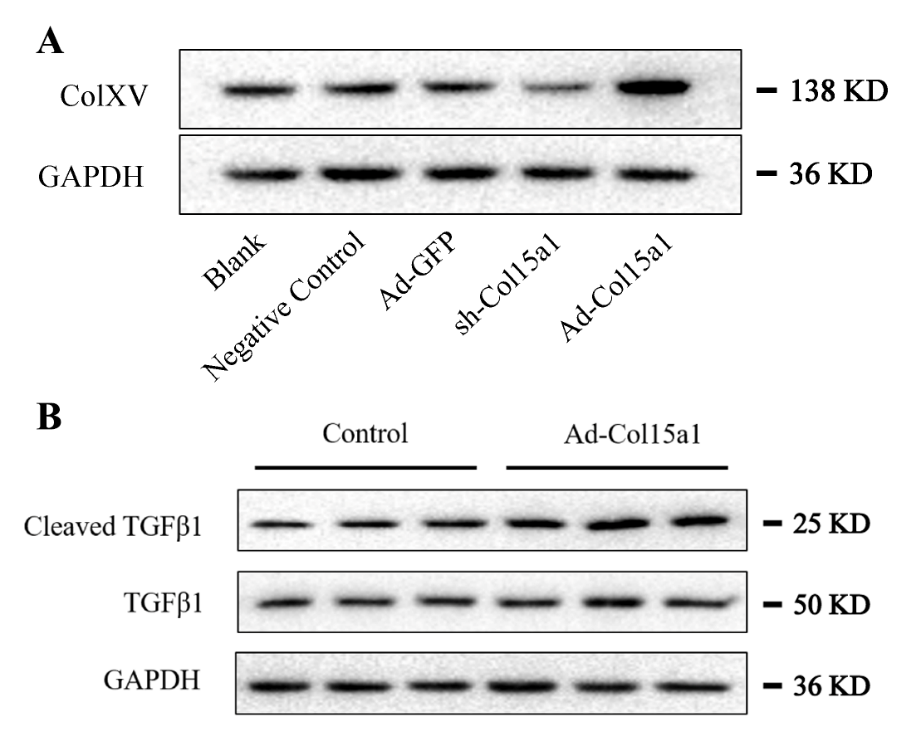


, Figure S1: Carrier expression efficiency and influence analyses.





Figure S2. Z-stack analysis and three-dimensional reconstruction
