# Supplementary figures and images for: ColXV Aggravates Adipocyte Apoptosis by Facilitating Abnormal Extracellular Matrix Remodeling in Mice
Source: Int J Mol Sci. 2020 Jan 31;21(3):959. doi: 10.3390/ijms21030959 (PMC7037489; doi:10.3390/ijms21030959)

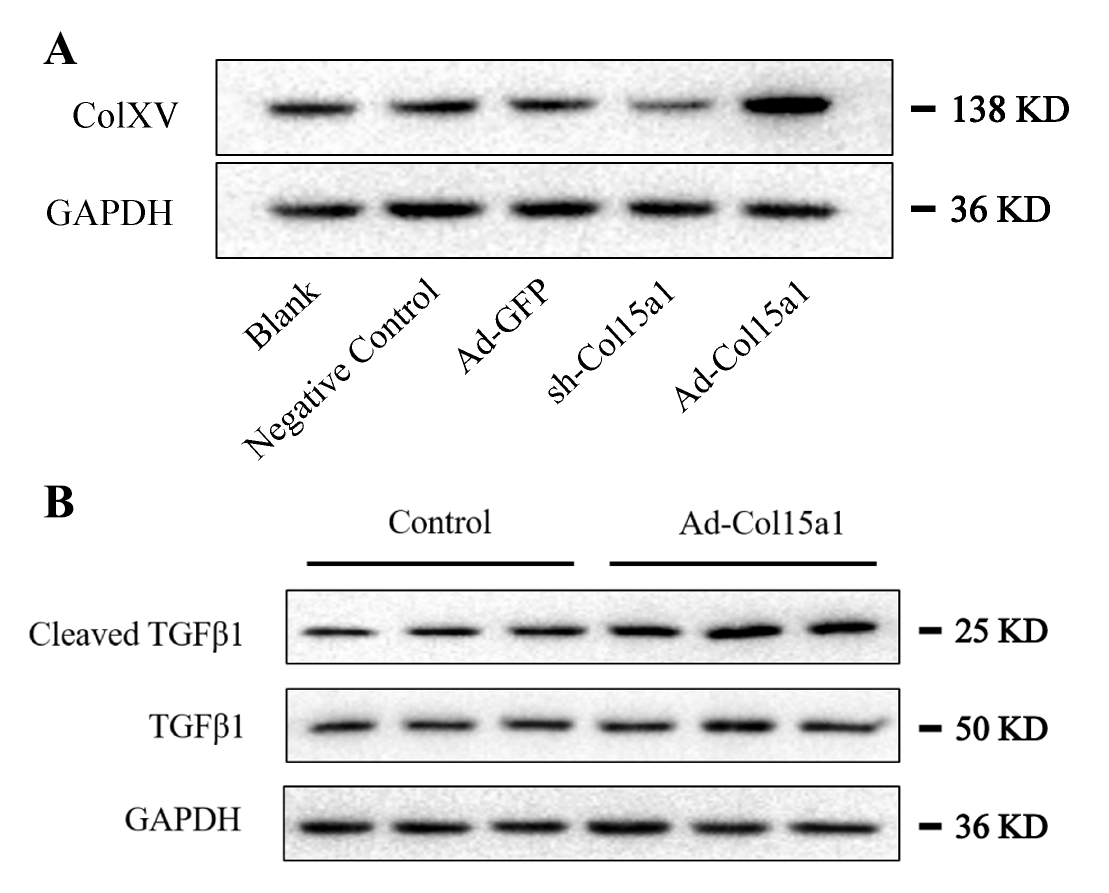

Supplement: Supplementary file 1 [file ijms-21-00959-s001.zip › Supplementary pictures/Figure S1.tif]

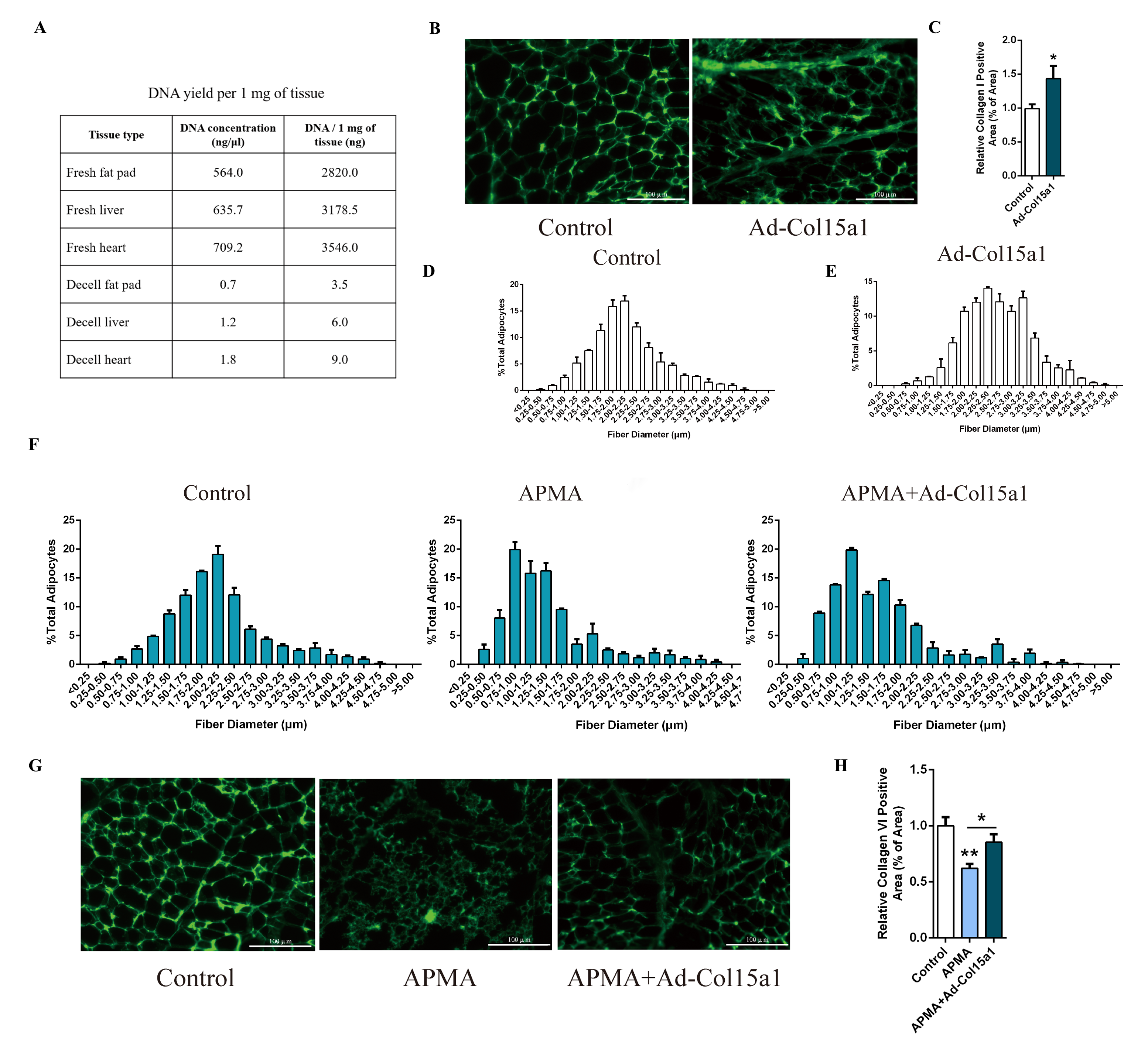

Supplement: Supplementary file 1 [file ijms-21-00959-s001.zip › Supplementary pictures/Figure S2.tif]
